# Effectiveness of human immunodeficiency virus prevention strategies by mapping the geographic dispersion pattern of human immunodeficiency virus prevalence in Nanning, China

**DOI:** 10.1186/s12889-024-18345-9

**Published:** 2024-03-16

**Authors:** Ping Cen, Guo Xu, Jianxun Wu, Jiao Qin, Jinfeng He, Xiaofang Deng, Xi Yang, Peng Lu, Mengni Nong, Junjun Jiang, Li Ye, Hongyang Tang, Bingyu Liang, Hao Liang

**Affiliations:** 1 Nanning Center for Disease Control and Prevention, Nanning, 530023 Guangxi China; 2 Guangxi Key Laboratory of AIDS Prevention and Treatment, School of Public Health, Guangxi Medical University, Nanning, 530021 Guangxi China; 3 Collaborative Innovation Centre of Regenerative Medicine and Medical BioResource Development and Application Co-constructed by the Province and Ministry, Life Science Institute, Guangxi Medical University, Nanning, 530021 Guangxi China; 4 Nanning Survey and Design Institute Group Co., Ltd., Nanning, 530022 Guangxi China

**Keywords:** The Guangxi AIDS conquering project, Geospatial approach, Heterogeneous spatial distribution, Human immunodeficiency virus, Acquired immunodeficiency syndrome

## Abstract

**Background:**

The Guangxi government initiated two rounds of the Guangxi AIDS Conquering Project (GACP) in 2010 (Phase I) and 2015 (Phase II) to control human immunodeficiency virus (HIV)/acquired immunodeficiency syndrome (AIDS) epidemics. However, the effectiveness of GACP in HIV prevention and treatment has rarely been reported. This study aimed to assess the effectiveness of the GACP implemented in Guangxi, China and provide data for strategy and praxis improvements to achieve Joint United Nations Programme on HIV/AIDS (UNAIDS) 95-95 targets.

**Methods:**

We used spatial approaches to trace the spatiotemporal distribution properties, epidemic trends, and correlation between macroscopic factors and HIV incidence using data from the Chinese HIV/AIDS case reporting system to explore the effects of the GACP.

**Results:**

During the GACP era, the HIV epidemic stabilized in urban centers, showing a downward trend in the Hengzhou and Binyang Counties in the eastern region, whereas it continued to increase in rural areas of the northwest region, such as the Long’an, Mashan, Shanglin, and Wuming Districts. The linear directional mean (LDM) of HIV infection reported cases displayed a southeast–northwest direction, with an LDM value of 12.52°. Compared with that in Phase I, Hengzhou withdrew from the high-high clustering area, and the west–north suburban counties pulled out the low-low clustering area during Phase II. Significant HIV clusters were identified in the eastern region during Phase I, whereas these clusters emerged in the northwestern areas during Phase II. Regarding HIV, socioeconomic status, population mobility, and medical care levels were the key social drivers of heterogeneous spatial distribution.

**Conclusions:**

The GACP assisted in effectively managing the HIV epidemic in urban and eastern areas of Nanning City. However, prevention and control efforts in rural regions, particularly those located in the northwest, may not have yielded comparable outcomes. To address this disparity, allocating additional resources and implementing tailored intervention measures for these rural areas are imperative.

**Supplementary Information:**

The online version contains supplementary material available at 10.1186/s12889-024-18345-9.

## Background

Human immunodeficiency virus (HIV) infection is a global health concern. Globally, the number of people living with HIV was 37.7 million, with 1.5 million people newly infected with HIV, in 2020 [1]. This suggests continued HIV transmission despite a reduction in its incidence. In China, the acquired immunodeficiency syndrome (AIDS) epidemic has been categorized into three phases: sporadic cases (1985–1988), endemic outbreaks (1989–1994), and the expansion phase (1995–present day) [2]. In October 2020, 1.045 million people in China were living with HIV/AIDS (prevalence: 0.075%) [3]. Driven by its policy, China has played a key role in the global fight against HIV/AIDS [4]. Nonetheless, the country faces complex challenges endeavoring to control the disease in both rural and urban areas [5]. Guangxi ranks third in terms of HIV infection cases in China, with the local population infection rate of 0.13% [6]. Nanning is ranked as the top city in Guangxi based on the number of reported surviving cases [7], with 7068 deaths from 2001 to 2020 [8]. Concerning the AIDS epidemic, the Guangxi government initiated the first (Phase I) and second (Phase II) rounds of the Guangxi AIDS Conquering Project (GACP) in 2010 and 2015, respectively [9, 10]. In the GACP, the local government established an AIDS Working Committee as the technical body responsible for oversight in 2010, to implement the responsibility of community leaders in AIDS prevention and treatment and hold those who fail to fulfill their responsibilities accountable. Combining local initiatives with the China Comprehensive AIDS Response (CARES) Programme, the Guangxi government has actively utilized investments in financial and human resources to enhance HIV prevention education, voluntary counseling and testing, sentinel surveillance, behavioral interventions, health care, and antiretroviral therapies (ARTs). However, the effects of GACP have rarely been evaluated or reviewed.

Spatial models help assess prevention efforts [11], plan resource allocation [12], infer gaps in service delivery, and understand biases in surveillance data, adapt services, and target interventions [13]. In sub-Saharan Africa, strategies for eliminating HIV are designed by mapping the geographic dispersion patterns of HIV-infected individuals [11]. Compared with the individual level, the spatiotemporal level of HIV-related outcomes can potentially provide fresh insights into public health strategies and prevention measures [14]. Although geographic information systems have been used for several years to monitor infectious diseases, their capacity to target services for regional disease prevention and control strategies is underutilized [13, 15].

This study aimed to assess the effectiveness of the GACP implemented in Guangxi, China, and provide data in strategy and practice improvements to achieve the UNAIDS 95-95 targets. These targets aim to ensure that, by 2025, 95% of individuals living with HIV are aware of their HIV status, 95% of those who know their HIV-positive status are receiving treatment, and 95% of individuals on HIV treatment have suppressed viral loads. We used a geostatistical framework to reveal the geographic dispersion pattern of HIV-infected individuals at different stages of GACP implementation to determine the effectiveness of GACP implementation.

## Methods

### Data sources

Data were acquired via the National HIV/AIDS Comprehensive Information System of China’s Disease Prevention and Control Information System [16]. To avoid or minimize duplicate case reporting, the local Center for Disease Control staff performed multiple logical checks on all completed case report forms by identifying numbers, names, addresses, and other relevant information to ensure data quality. The HIV/AIDS Case Reporting System (CRS) helped us obtain detailed information on each case and its geographic distribution. Population, economy, and healthcare data were obtained from the Nanning Statistical Yearbook of the Nanning Statistical Bureau.

### Data administration

All HIV/AIDS cases identified in the CRS from 1996 to 2021 in Nanning were evaluated. Participants with conflicting addresses were excluded from the study. All reported cases were geocoded according to street address records at city and county levels to acquire valid latitude and longitude coordinates for the present address.

### Time slicing

The study period was categorized into three distinct phases: before the GACP period (1996–2009), the first GACP period (Phase I, 2010–2015), and the second GACP period (Phase II, 2016–2021). In 2010, the Guangxi government launched the first phase of GACP in response to the HIV epidemic [9]. We evaluated the shift in the spatial distribution of reported HIV infection cases in Nanning over a 25-year period using data before and after the implementation of GACP as a real-world database.

### Spatial cluster analysis

We performed spatial analyses based on the total number of individuals with HIV in the Nanning area for each period. First, the district boundaries were linked to local HIV-reported cases for plot visualization, and the geographic clustering of HIV in the region for each period was tested using Moran’s *I* index [17]. Adjacent regions were defined as neighbors, and a spatial weight matrix was used to clarify the spatial relationships among districts. Negative, positive, and zero values of Moran’s *I* indicated overdispersed, clustered, and spatially random distributions, respectively [18]. Next, we used local indicators of spatial association (LISA) to determine exactly where local clustering occurred. Regions were divided into different cluster levels based on the significance of LISA statistics [19]. Finally, we generated spatial distribution maps using ArcMap software version 10.5.0.6491 (ESRI, Redlands, CA, USA).

### Centroid-transferring curve model

The centroid-transferring curve model was constructed to trace spatiotemporal pattern changes in HIV infection cases in Nanning by identifying centroids and their shifts. Here, we measured the mean centers (MCs) of the locations of HIV infection by recognizing the geographic center (or center of concentration) for a set of points [20]. Determining the average x- and y-coordinates of MCs was beneficial for tracking changes in the distribution or comparing the distributions of different types of features. A standard deviational ellipse (SDE) typically indicates the spatial distribution of a set of point locations [21]. Therefore, we employed this visualization technique to effectively quantify the distribution and orientation of data points within multiple dimensions for HIV/AIDS cases location, accurately indicating their variability and dispersion. Moreover, the Linear Directional Mean (LDM) represented the average orientation of all lines in the locations of HIV/AIDS cases, providing a precise measure of their collective trend. Additionally, the LDM could comprehensively evaluate variations in line lengths and geographic centers [21]. In this study, annual MC movements from 1996 to 2021 were used to calculate the LDM. These LDMs demonstrated an annual LDM trend during the study period. The average LDM over the past 25 years indicated the overall trend of LDM.

### Spatiotemporal scanning

Spatial cluster analysis of HIV-1 case reporting was implemented using SaTScan [22]. Statistically significant windows were evaluated using Monte Carlo simulations; the primary and secondary clusters were determined based on window likelihood values [23]. In this study, the relative risk (RR) was calculated using the observed number of individuals within the windows [24]. Statistically significant clusters in the scanning window radius, observed and expected number of positive cases within the circle, log-likelihood ratio, and *p value* were used for analysis and mapping [25]. Significant clusters obtained using SaTScan version v10.1, July 2022 (The SaTScan software was developed by Martin Kulldorff together with Information Management Services Inc.) were mapped using ArcMap 10.5.0.6491 (ESRI, Redlands, CA, USA).

### Geographical and temporal weighted regression model

As an extension of the geographically weighted region model, geographical and temporal weighted regression (GTWR) considers the spatial non-stationarity of geographic data and incorporates temporal effects into the model calculation, thus improving its goodness-of-fit [26]. The GTWR is expressed as follows:

$${Y}_i={\beta}_0\left({u}_i,{v}_i,{t}_i\right)+\sum_{k=1}^K{\beta}_k\left({u}_i,{v}_i,{t}_i\right){X}_{ik}+{\varepsilon}_i$$

Unlike fixed-coefficient global regression models, GTWR allows parameter estimates to vary both spatially and temporally. Therefore, this method can simultaneously capture spatiotemporal variations [26].

## Results

### Distribution of HIV infection cases

From 1996 to 2021, 25,868 HIV/AIDS cases were confirmed and reported to the CRS in Nanning. Among these, 13,909 and 11,959 patients had AIDS and HIV infections, respectively. Of these, 2216 (8.57%) were excluded because of inconsistent addresses at the county/district level. Thus, data of 23,652 patients (91.43%) was included in the final analysis.

**Fig. 1:**
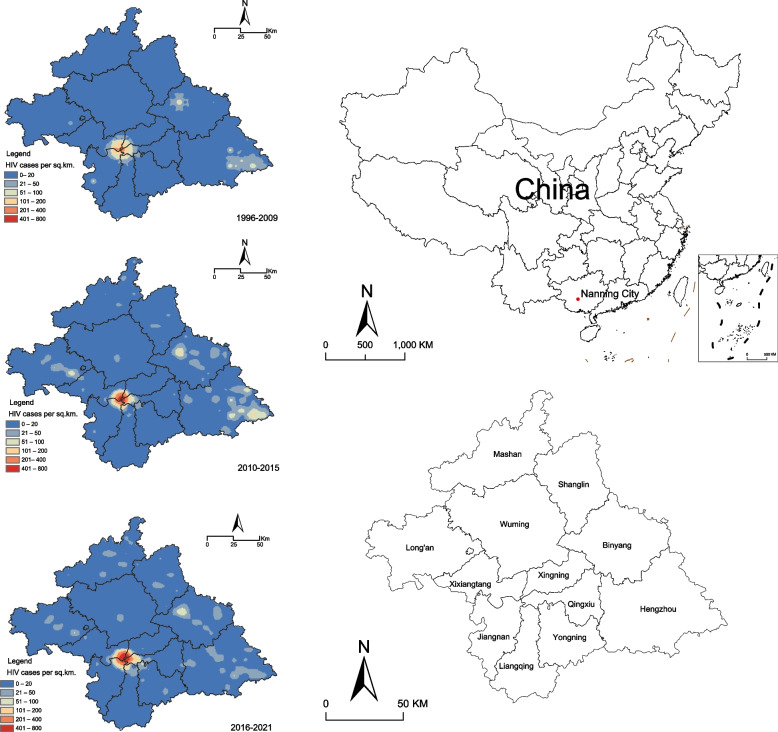
The distribution by place of the human immunodeficiency virus (HIV) in Nanning during different periods. The signal of the red point represents the site of Nanning city in China. Colors represent local HIV prevalence. Blue represents low HIV prevalence, whereas red represents high HIV prevalence. The shapefile for China was downloaded from the standard map service (http://bzdt.ch.mnr.gov.cn/) (drawing review no. Das, Li, Allston, and Kharfen [12] 1822), from which the Nanning shapefile was extracted using ArcGIS

The spatial distribution of HIV infection cases reported in Nanning showed the highest number of cases in the central region during the entire period of 1996–2021 (Fig. 1). HIV distribution in Nanning gradually expanded over time. Prior to the GACP, most reported HIV/AIDS cases were concentrated in downtown commercial areas, followed by the eastern part of Nanning District. Thereafter, during the first GACP period, the reported cases spread to suburban counties, particularly the Binyang and Hengzhou Counties in the east and Long’an County in the west. During the second GACP period, HIV prevalence in urban centers stabilized. In contrast, the prevalence decreased in the east, such as in the Binyang and Hengzhou Counties, and increased continuously in the north, including in the Mashan and Shanglin Counties.

**Fig. 2:**
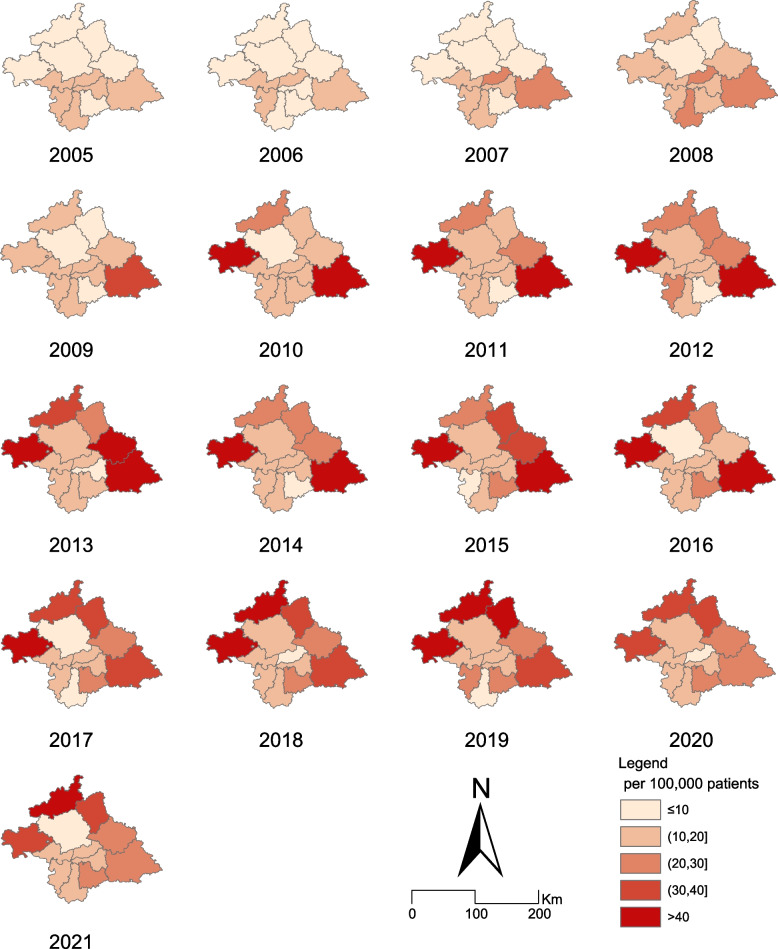
Choropleth maps of incidence of acquired immunodeficiency syndrome/human immunodeficiency virus (per 100,000) at the county level. The finding is shown for Nanning, China, from 2005 to 2021. The shapefile for China was downloaded from the standard map service (http://bzdt.ch.mnr.gov.cn/) (drawing review no. Das, Li, Allston, and Kharfen [12] 1822), from which the Nanning shapefile was extracted using ArcGIS

Although Fig. 1 shows concentrated areas with reported HIV/AIDS cases, owing to regional differences in population characteristics, we further observed the spatiotemporal distribution of AIDS infection rates. All areas of Nanning City reported HIV infection cases in 2005 (Fig. 2). Thereafter, the annual number of HIV screenings in Nanning City has increased from 484,324 in 2006 to 2,426,490 in 2021 (see Additional file 1). An overall increasing trend of HIV incidence was noted in all regions but with a strong spatiotemporal inhomogeneous distribution. In contrast to urban centers, where the incidence stabilized, the incidence increased faster in urban suburbs. Among them, Hengzhou and Binyang Counties showed an increase, followed by a decrease in HIV/AIDS incidence, whereas Yongning District and Mashan and Shanglin Counties showed a steady increase (Fig. 2).

### Spatial cluster analysis

The global Moran’s *I* value indicated that the HIV infection rate clustered in 1996–2009, 2010–2015, and 2016–2021 (*p* <  0.05), suggesting local clustering in each period (Table 1).

**Table 1:** Spatial autocorrelation analysis of annual human immunodeficiency virus infection at different periods in Nanning, China

Year	Moran’s *I*	*Z* value	*P* value
1996–2009	0.443018	10.836907	< 0.001
2010–2015	0.235530	5.762650	< 0.001
2016–2021	0.733795	17.357180	< 0.001

**Fig. 3:**
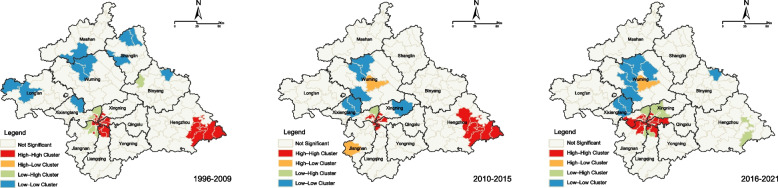
Local indicators of spatial association cluster maps of the distribution of human immunodeficiency virus. These are cases reported in Nanning, China, from 1996 to 2021

The initial phase of GACP implementation may have changed the correlation or divergence in HIV transmission across different regions, leading to a lower global Moran’s *I* index for phase I compared with the pre-GACP era. The LISA cluster map identified local clustering areas of HIV infection in the Nanning Region by showing five geographical clustering types: high-high, low-low, high-low, low-high, and not significant. We focused on regions with significantly high-high clustering, where regions showing high HIV infection rates were surrounded by other regions with high HIV infection rates. Regarding the entire period, we also found a high clustering of HIV infections in downtown commercial neighborhoods spanning five central districts. Post-2015, the geographic area of high-high clustering started spreading from the center to the periphery. In addition, before the GACP period and in the first GACP period, Hengzhou County east of Nanning was also observed as a high-high cluster. However, during the second GACP period, this county withdrew from the high-high cluster area (Fig. 3) (see Additional file 2).

### Centroid movement analysis

**Fig. 4:**
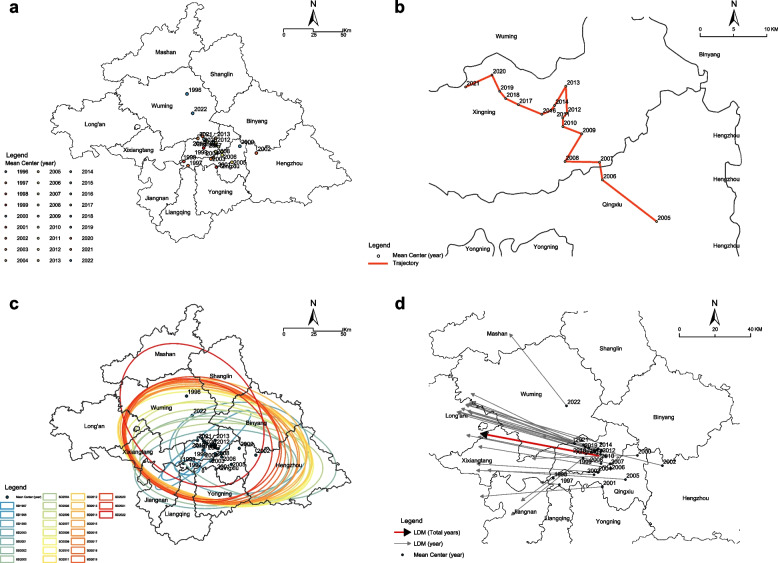
Centroid movement path of the acquired immunodeficiency syndrome (AIDS) incidence. The findings are reported in Nanning from 1996 to 2021. AIDS mean center (**a**, **b**), standard deviational ellipse (**c**), and linear directional mean (**d**) from 1996 to 2021. Dots indicate mean center (MC) of cases per year. Ellipses represent standard deviational ellipse (SDE) of cases per year. Gray arrow indicating the Linear Directional Mean (LDM) of cases per year. Red arrows illustrate the overall Linear Directional Mean (LDM) of cases spanning from 1996 to 2022

We estimated the shift directions and distances of the centroids of HIV infection cases reported in Nanning from 1996 to 2021 based on the results of the spatial pattern simulations (Fig. 4a, b) (see Additional file 3). Based on the overall distribution of MCs, the HIV-reported case centroids were mainly concentrated in the central district of Nanning, which is consistent with the geometrical center of the study area. Before 2004, there were no reports of HIV infection cases in some areas presenting as imported or sporadic cases in the early stages, and local epidemics were predominant. Consequently, the SDE was smaller, and no evident pattern was noted in the case reports. Overall, from 2005 to 2021, an evident trend was noted, in that the centroid of the HIV-reported case area displayed a southeast–northwest direction, with an orientation of 39° and a shift distance of 33.23 km (Fig. 4c). This period was divided into three stages. Before the GACP period, most SDEs emerged at similar locations with the same shape, size, and in the East-West direction. They appeared primarily in 9 of the 12 prefectures and districts, except for the Long’an, Mashan, and Shanglin Counties. In the first GACP period, the SDEs shifted northwest, and the centroid shifted 5.51 km in the west-by-north. In the second GACP period, the centroid shifted by 12.31 km from west to north.

The direction of disease diffusion and movement of the annual LDM locations were examined (Fig. 4d). Most LDMs were directed toward the west. The LDM trend indicated a west-by-north direction with an orientation of 12.52°. This result indicates the spread of HIV in the study region.

### Spatiotemporal scanning

**Fig. 5:**
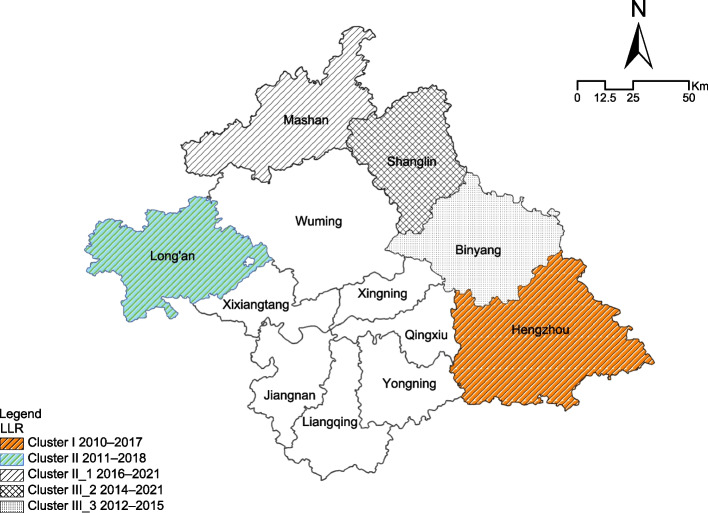
Spatiotemporal scanning aggregation analysis of acquired immunodeficiency syndrome incidence. The findings are reported in Nanning, China, from 2005 to 2021

Five non-overlapping statistically significant clusters were identified. The most likely cluster, i.e., Cluster 1, was observed in Hengzhou County from 2010 to 2017. The RR was 2.41 (*p* < 0.001), implying that people in the cluster had a 141% higher risk of HIV infection than did those outside the cluster. From 2011 to 2018, Long’an County was classified as secondary Cluster 2. An RR of 2.41 (*p* < 0.001) was estimated, indicating that individuals living at a location within this cluster were 2.41 times more likely to be infected with HIV compared with those living outside the cluster. The first, second, and third tertiary clusters (RR = 2.04, 1.61, and 1.68, respectively; *p* < 0.001) included the Mashan (2016–2021), Binyang (2012–2015), and Shanglin (2014–2021) Counties, respectively (Fig. 5) (see Additional file 4).

### Macroscopic factor-associated analysis

**Fig. 6:**
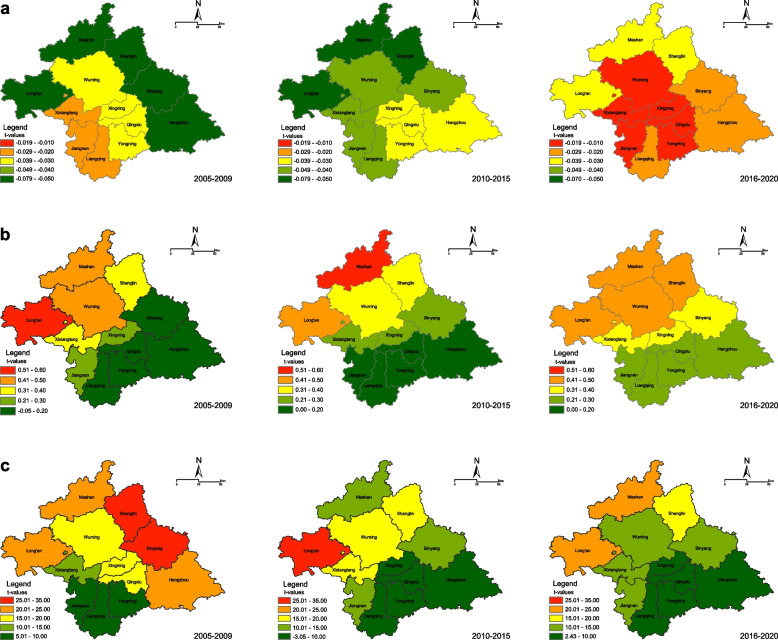
Spatial distribution of the average coefficients for acquired immunodeficiency syndrome/human immunodeficiency virus (AIDS/HIV). These are macroscopic factors at three different stages. Spatial mapping of coefficients and corresponding t-values. The dependent variable was the HIV-reported cases in Nanning from 2005 to 2020. **a** Gross domestic product coefficients and t-values, **b** per capita expenditure for healthcare and t-values, and **c** mechanical growth of population and t-values

The GTWR model was used to simulate the spatiotemporal relationship between macroscopic factors and HIV prevalence (Fig. 6). The fitting results of the GTWR exhibited stable performance; the adjusted *R*^2^ and corrected Akaike information criterion were 0.807 and 298.971, respectively. The AIDS/HIV macrofactor coefficients exhibited different spatial distribution patterns in the three epochs. The average coefficient of Gross Domestic Product (GDP) for Nanning’s administrative district was negative, ranging between − 0.016 and − 0.079, with a gradual decrease from the city center to the surrounding counties, suggesting that economically underdeveloped areas had higher HIV/AIDS rates (Fig. 6a). An increase in health expenditure per capita was positively associated with an increase in HIV infection rates in all regions, with the coefficient value increasing gradually from southeast to northwest. This indicates that HIV infection rates are higher in districts with higher per capita health expenditures compared with those with lower per capita health expenditures (Fig. 6b). A strong positive relationship was noted between the migrant population and HIV infection rates. During the second GACP period, although the population mobility coefficient of the central and eastern regions declined considerably, that of the northwestern regions, including Long’an, Mashan, and Shanglin, remained elevated (Fig. 6c).

## Discussion

This study evaluated the effectiveness of GACP by mapping the geographic dispersion pattern of HIV prevalence. Since the GACP was initiated, the HIV epidemic has been contained in Nanning, suggesting that the interventions have achieved remarkable results. However, the HIV epidemic has shifted toward diffusion in the surrounding rural counties. Economic development, floating population, and medical care levels may be driving factors for the spatial heterogeneity of AIDS.

Visualization of the habitual residence of HIV-reported cases in Nanning over a 25-year period revealed that the distribution of cases became more geographically scattered. The city center has become a hub for HIV-reported cases with a stable HIV prevalence. However, HIV prevalence in the Hengzhou and Binyang Counties decreased during the second GACP period. Furthermore, the LISA analysis indicated that Hengzhou County withdrew from the high-high clustering region, indicating that the two phases of the GACP played a big role in HIV control in these two counties. Hengzhou County was the hardest-hit area, followed by Binyang County and the urban center where the first HIV-reported case was detected [8]. During the GACP period, local governments stepped up multisectoral collaborations to expand HIV testing coverage, promote HIV education, and combat sex trafficking based on the CARES policy [27]. HIV awareness, testing, and treatment coverage have also improved.

Nevertheless, some northwestern administrative regions, including Long’an County, Mashan County, and Shanglin County, have experienced an increasing HIV prevalence during the GACP period. Furthermore, scanning statistics identified five HIV clusters in border counties after 2010. In the second phase of the GACP, tertiary clusters appeared in the Mashan and Shanglin Counties. Our results confirmed that the HIV epidemic in Nanning was not ubiquitous but was an evident geographically distinct cluster, with a disproportionately high number of reported HIV infection cases. Notably, in northwestern suburban counties, HIV/AIDS prevention was only completely implemented after launching the GACP, compared with other areas that implemented the CARES policy before launching the GACP. The number of HIV tests performed has increased annually during the GACP period, which could have played a vital role in increasing the incidence of HIV infection. However, the detection of HIV clusters implies that current interventions may be suboptimal in these districts. HIV clusters were mainly distributed in the northwestern counties of Nanning City, likely reflecting the accumulation of specific spatiotemporal risk factors [28, 29]. Disease control and prevention departments should pay close attention to epidemic hotspots, focusing on strengthening HIV/AIDS surveillance and prevention in the surrounding rural areas in the northwest.

Subsequently, we tracked the spatiotemporal variation in the reported HIV infection cases in Nanning by identifying centroids and their shifts. The results also showed an evident trend: the centroids of the HIV-reported case area displayed a southeast–northwest direction, specifically in Phase II of the GACP, suggesting that Nanning City might experience a diffusion of HIV outward from cities into rural areas, consistent with other countries and globally [23, 30]. There are several possible reasons for this shift in spatiotemporal centroids. First, given the historically higher HIV prevalence in the southeast, several HIV prevention and treatment programs launched by the GACP have focused on this region, which has led to HIV epidemic stabilization or even a decline. Second, since the GACP was initiated in 2010, older HIV-infected people have been detected in northwestern rural Nanning. However, effective interventions targeting this population are lacking, resulting in a continuous increase in infection rates [31]. Third, changes in proximity to urban connectivity may also play a role. Frequent border population movement between northwestern counties and cities with an elevated incidence of AIDS, including Congzuo, Baise, Hechi, and Laibin, may increase the incidence of AIDS [32, 33].

This study explored the spatial characteristics and factors correlating with HIV transmission. The effects of different macroscopic factors on the AIDS epidemic were more prevalent in northwestern Nanning than in southeastern Nanning, as evidenced by the higher estimated coefficients. First, all regression coefficients of economic development were negative, consistent with previous findings related to the spatial distribution of AIDS and the main socioeconomic driving factors in China [34, 35]. Low-cost sex trafficking [27] and poverty [36, 37] in northwest Nanning have been proven to exacerbate AIDS/HIV infection. Second, our findings confirmed that the floating population positively affected the number of HIV infection cases in Nanning. Previous studies have linked migration and mobile populations to an increased risk of HIV [28, 38], which may have contributed to the AIDS epidemic in northern Nanning [11]. Furthermore, lower per capita medical expenditure was associated with higher rates of HIV infection. In contrast, counties in rural areas are significantly more likely to lack access to medical investment [11, 39], and medically underserved areas may restrict the surveillance capability of spatial epidemiology [34, 40]. Therefore, health departments should consider adopting effective HIV prevention allocation strategies in rural areas with insufficient economic and health resources.

This study has some limitations. First, we cannot claim causality between economic and demographic variables or other social phenomena and HIV transmission. Second, the multifaceted nature of factors influencing reported HIV/AIDS cases, such as potential underreporting, may have influenced hotspot identification in our study. This could potentially limit a comprehensive and accurate assessment of the true effectiveness of efforts in preventing and controlling HIV. However, the existing data did not allow us to adjust for these factors. Third, some critical ecological variables were not included in our geospatial models, which may have affected the validity of the prediction maps. Despite these limitations, the findings of this study highlight the advantages of developing geospatial technologies to improve disease mapping and surveillance. Importantly, we identified trends in the spatial distribution of HIV epidemics following the implementation of local prevention strategies. This finding will benefit future targeted HIV prevention and control efforts in Guangxi Province.

## Conclusions

The HIV epidemic has been effectively controlled, particularly in areas with higher HIV prevalence, suggesting that the GACP has achieved remarkable results. However, with the implementation of the GACP, HIV diffusion shifted to the surrounding rural counties. This would exacerbate the significant urban–rural disparities in medical health care. Thus, in the third stage of the GACP, policymakers should pay more attention to the upscaling of age-specific HIV education and testing to initiate early ART in rural areas. Additionally, more resources and appropriate interventions should be committed to subpopulations and regions that are underserved and require health facilities and care personnel in the country.

### Supplementary Information

**Supplementary Material 1.**

**Supplementary Material 2.**

**Supplementary Material 3.**

**Supplementary Material 4.**

## Data Availability

The datasets used and/or analysed during the current study are available from the corresponding author on reasonable request.

## Abbreviations

AIDS: Acquired immunodeficiency syndrome
CRS: Case Reporting System
GACP: Guangxi AIDS Conquering Project
GTWR: Geographical and temporal weighted regression
HIV: Human immunodeficiency virus
LDM: Linear directional mean
LISA: Local indicators of spatial association
MCs: Mean centers
RR: Relative risk
SDE: Standard deviational ellipse
GDP: Gross Domestic Product
UNAIDS: Joint United Nations Programme on HIV/AIDS
