# Supplementary material for: Effectiveness of human immunodeficiency virus prevention strategies by mapping the geographic dispersion pattern of human immunodeficiency virus prevalence in Nanning, China
Source: BMC Public Health. 2024 Mar 16;24:831. doi: 10.1186/s12889-024-18345-9 (PMC10944615; doi:10.1186/s12889-024-18345-9)
Supplement: Supplementary file 1 — Supplementary Material 1. [file 12889_2024_18345_MOESM1_ESM.pdf]

Additional file 1

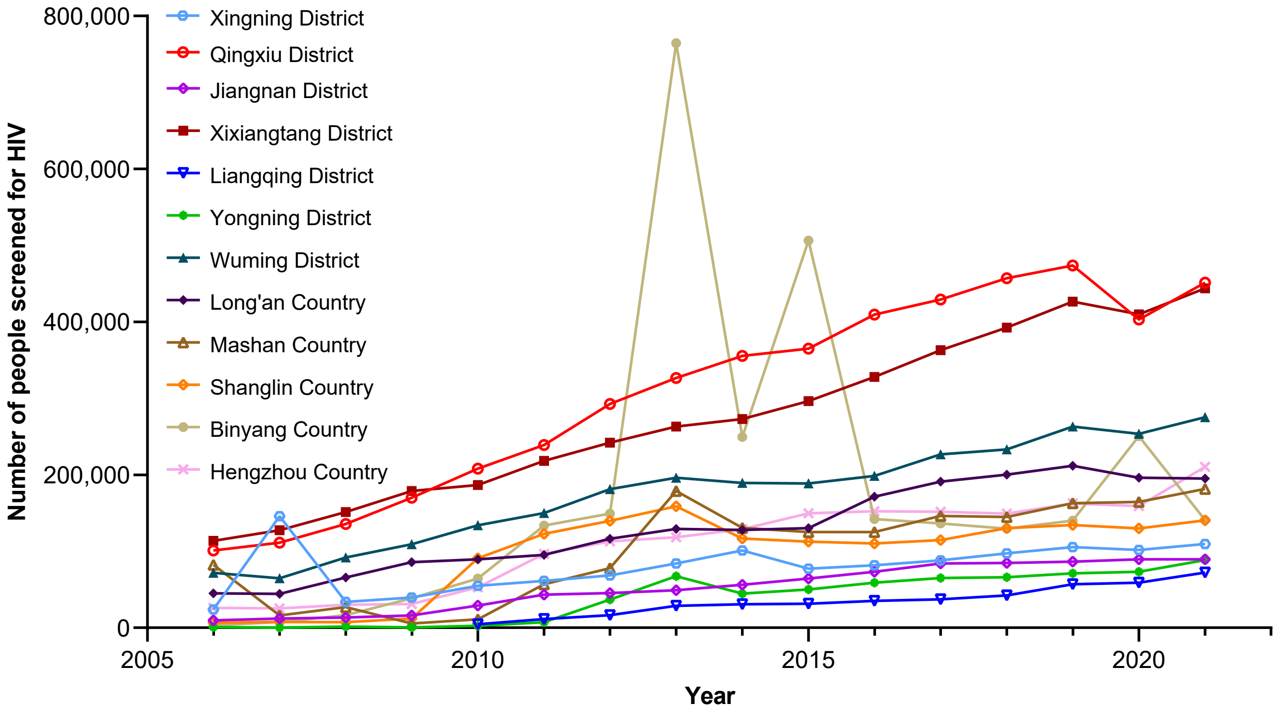

Figure S1. The annual number of people screened for human immunodeficiency virus (HIV) infection in counties and districts of Nanning City from 2006 to 2021.
