# Supplementary material for: Effectiveness of human immunodeficiency virus prevention strategies by mapping the geographic dispersion pattern of human immunodeficiency virus prevalence in Nanning, China
Source: BMC Public Health. 2024 Mar 16;24:831. doi: 10.1186/s12889-024-18345-9 (PMC10944615; doi:10.1186/s12889-024-18345-9)
Supplement: Supplementary file 2 — Supplementary Material 2. [file 12889_2024_18345_MOESM2_ESM.pdf]

Additional file 2

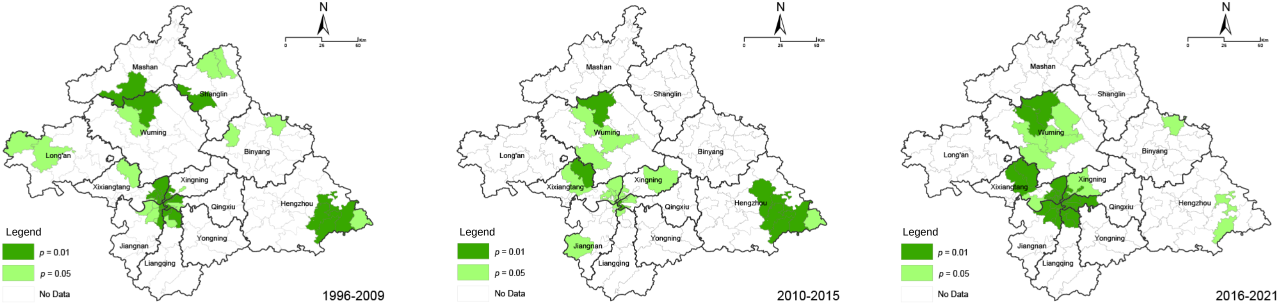

Figure S2. Significance test results of the local spatial autocorrelation of human immunodeficiency virus reported cases in Nanning, China, from 1996 to 2021.
