# Supplementary material for: Effectiveness of human immunodeficiency virus prevention strategies by mapping the geographic dispersion pattern of human immunodeficiency virus prevalence in Nanning, China
Source: BMC Public Health. 2024 Mar 16;24:831. doi: 10.1186/s12889-024-18345-9 (PMC10944615; doi:10.1186/s12889-024-18345-9)
Supplement: Supplementary file 3 — Supplementary Material 3. [file 12889_2024_18345_MOESM3_ESM.docx]

**Additional File 3**

**Table S1.** Calculation results of the centroid movement path method of AIDS incidence rate in Nanning from 1996 to 2021

| Year | Displacement distance (km) | Mobile angle | | Moving direction |
| --- | --- | --- | --- | --- |
| 1996 |  |  |  |  |
| 1997 | 60.41 | 1.564870562 | 89°39′37.72″ | West by south |
| 1998 | 4.80 | –0.861114356 | 50°39′42.41″ | East by south |
| 1999 | 19.10 | 0.611804794 | 35°3′13.8″ | East by north |
| 2000 | 28.25 | 0.039054274 | 2°14′15.52″ | East by north |
| 2001 | 25.36 | 0.754261947 | 43°12′57.69″ | West by south |
| 2002 | 33.35 | 0.348525945 | 19°58′8.64″ | East by north |
| 2003 | 35.85 | 0.125178785 | 7°10′19.98″ | West by south |
| 2004 | 2.38 | 0.544546976 | 31°12′0.88″ | East by north |
| 2005 | 14.89 | –0.287705042 | 17°30′56.58″ | East by south |
| 2006 | 9.76 | –0.706649668 | 41°30′43.04″ | West by north |
| 2007 | 2.67 | –1.439619488 | 83°30′57.17″ | West by north |
| 2008 | 4.78 | –0.037991492 | 3°49′23.69″ | West by north |
| 2009 | 4.74 | 1.044572557 | 59°50′58.56″ | East by north |
| 2010 | 2.83 | –0.409544665 | 24°32′5.35″ | West by north |
| 2011 | 1.29 | 1.560553163 | 89°24′47.2″ | East by north |
| 2012 | 0.91 | 0.866347243 | 49°38′16.95″ | East by north |
| 2013 | 4.04 | –1.548001005 | 89°18′21.87″ | West by north |
| 2014 | 3.27 | 1.057473343 | 60°35′19.53″ | West by south |
| 2015 | 0.93 | 0.956284017 | 54°47′27.74″ | West by south |
| 2016 | 1.34 | 0.400918292 | 22°58′15.33″ | West by south |
| 2017 | 3.56 | –0.430884188 | 25°18′43.76″ | West by north |
| 2018 | 1.99 | –0.498403177 | 29°26′36.97″ | West by north |
| 2019 | 1.36 | –0.92955368 | 54°44′25.79″ | West by north |
| 2020 | 2.66 | –1.177887031 | 68°30′43.36″ | West by north |
| 2021 | 4.07 | 0.440953306 | 25°15′53.15″ | West by south |

AIDS, acquired immunodeficiency syndrome
