# Supplementary material for: Effectiveness of human immunodeficiency virus prevention strategies by mapping the geographic dispersion pattern of human immunodeficiency virus prevalence in Nanning, China
Source: BMC Public Health. 2024 Mar 16;24:831. doi: 10.1186/s12889-024-18345-9 (PMC10944615; doi:10.1186/s12889-024-18345-9)
Supplement: Supplementary file 4 — Supplementary Material 4. [file 12889_2024_18345_MOESM4_ESM.docx]

**Additional File 4**

**Table S2.** Spatiotemporal scanning aggregation analysis of AIDS incidence in China from 1996 to 2021

| Spatial clusters | County | Year | Number of cases | Expected cases | *RR* | *Log likelihood ratio* | *P value* |
| --- | --- | --- | --- | --- | --- | --- | --- |
| Most likely cluster | Hengzhou | 2010–2017 | 3201 | 1434 | 2.41 | 869.66 | < 0.001 |
| Secondary cluster | Long’an | 2011–2018 | 1187 | 505 | 2.41 | 341.23 | < 0.001 |
| Tertiary cluster 1 | Mashan | 2016–2021 | 941 | 471 | 2.04 | 186.15 | < 0.001 |
| Tertiary cluster 2 | Binyang | 2012–2015 | 1040 | 645 | 1.61 | 104.79 | < 0.001 |
| Tertiary cluster 3 | Shanglin | 2014–2021 | 966 | 583 | 1.68 | 107.94 | < 0.001 |

AIDS, acquired immunodeficiency syndrome; RR, relative risk
